# Books Review

**Published:** 1871-05

**Authors:** 


### Books Review.

*Memoirs of United States Sanitary Commission.*—Issued by U. S. Sanitary Commission. Published by HURD & HOUGHTON.

This is the title of an exhaustive and well written Report of the workings of the Sanitary Commission during our last war. The work is divided into Historical, Statistical and Surgical, and consists of four volumes, elegantly bound in cloth. The editorial department consists, as its name implies, of an elaborate sketch of the great system of the Society from the first moment of its organization, through the war to its close, being a faithful story of its experiences, showing what a great work of benificence it accomplished. The statistical is devoted principally to anthropological considerations concerning American soldiers, and is properly illustrated with charts, diagrams, etc. The last two volumes are reserved for the (to us at least) more important subject of Military Surgery, and certainly seem to be as complete an epitome of the question as could well be imagined. The views given are all of the most recent character, and the topics discussed show a vast amount of original research. Clinical illustrations are prominent features in the work, and serve to give a more thorough interest to the reader.

This volume of the Surgical Memoir comprises an analysis of four hundred and thirty-nine recorded amputations in the contiguity of the lower extremity, by Stephen Smith, M. D.; and investigations upon the nature, causes and treatment of Hospital gangrene, as it prevailed in the Confederate Armies in 1861—1865, by Joseph Jones, M. D. This paper by Dr. Jones is most beautifully illustrated by chromo-lithographic plates, showing the appearances, both general and microscopic, of parts involved in gangrene. These reports are very complete and instructive, constituting valuable chapters upon military surgery. The United States Sanitary Commission have contributed largely to humanity, and to sanitary, and medical, and surgical science.

---

*Causation, Course, and Treatment, of Reflex-Insanity in Women.* By H. R. STORER, M. D., L. L. B. Boston: LEE & SHEPARD. New York: LEE, SHEPARD & DILLINGHAM, 1870.

This is the republication in systematic form of a communication to the American Medical Association, in 1865, at its session in Boston, and presented in the Transactions of last year.

The following are the main propositions presented in the book, which are fully shown, and at the present time, we believe, recognized and accepted by the profession:

"1. That while the brain is undoubtedly the seat of insanity, yet it is not necessarily the seat of its cause.

"2. And that this is proved by *a priori* reasoning and both negatively and positively, by the results of autopsies.

"3. That while idiopathic insanity, requiring direct cerebral or simply moral treatment alone, is very rare, sympathitic, or reflex insanity, requiring treatment of a special character, is extremely common.

"4. And that, on one hand, such reflex causation is, and should be, much more common in females than in males; while, on the other hand, of the various forms of it occurring in females, the majority of them are owing to functional or organic diseases of the uterus and its appendages: in other words, that they are of a sexual character."

These several propositions are sustained by the testimony of a large number of Superintendents of Insane Asylums and others familiar with the insane.

The whole ground involved in these propositions is most ably discussed; and the absurdity of many of the opinions formerly entertained concerning the causes and treatment of insanity are amply shown. Some of these are: That insanity in woman is seldom dependant upon a pelvic cause; that thorough physical examinations of insane woman are not required; that they would be injurious to the patient herself, and, if in an asylum, to the other patients, and to general discipline, etc., etc.

This book, if carefully read by the profession, would do much towards directing attention to the causes of insanity, and thus, in many instances, lead to an earlier judicious treatment.

The chief merit of the paper is, that it is one of the first upon the subject, and was given to the profession at a time when the propositions were comparatively new to all, and violently opposed by many: written in the very beginning of a revolution which has now become complete.

---

*The Freeman Trial.* By David Dimon.

The case of Freeman, who murdered the VanNest family, is still fresh in the minds of the public, though some twenty years have passed since the committal of the act, and in this pamphlet we have a *resume* of the evidence evolved at this remarkable trial. Those who take an interest in cases involving "temporary insanity," may gain an idea from its perusal.

---

*Announcement of the Summer Session of Harvard Medical School.*

Embraces the usual amount of matter peculiar to Medical Colleges, with a schedule of recitations and lectures for the coming year.

---

*Report of Board of Trustees of Michigan Asylum for Insane, for the years* 1869-70.

Giving an account of the financial condition and management of affairs pertaining to this institution, with Report of Medical Superintendent concerning 543 cases treated; with what success a few tables of statistics make known. The report is of same interest to the Psychological inquirer.

---

*Anæsthetics.* By Edward R. Squibb.

A paper on the above subject, from the pen of Dr. Squibb, cannot be otherwise than valuable. He deals with his subject in a manner which expresses much study and research in the premises. We can assure our professional friends that it will repay perusal.

---

*Management of the Obstetrical Forceps.* By C. C. P. Clark, M. D.

The doctor enters the field in earnest, and gives us a not all-to-gether pleasing picture of the dangers and difficulties to be encountered in the use of the Forceps. He attributes them to be too often the result of the teachings of the manipulator, which, as he expresses it, is usually "defective and erroneous in substance, and in manner unnecessasily complex and obscure, rather than a defect in the instrument itself, them which the "whole armory of our art furnishes none calculated to be of more benefit in saving life and lessening suffering." In place of the usual instructions, he proposes to institute a set of rules which shall be simple, intelligible and in all places applicable to the emergency. They are ten in number, and differ somewhat more or less from those now in vogue. The paper affords, undoubtedly, some valuable matter for the consideration of all, and is certainly entitled to an earnest perusal.

*Eleventh Annual Report of Superintendent of State Lunatic Asylum for Insane Criminals.*

The report embraces the usual features of papers of the character, composed mainly of statistical matter, and not dealing with individual cases.

---

*Valedictory Address to Graduating Class of Rush Medical College, Class of* 1870–71. By Moses Gunn, M. D., Professor of Surgery.

A reprint from " Chicago Medical Journal," of the eloquent and instructive address at the last oommencement of Rush Medical College.

